# Afternoon Discharge Rounds for Capturing Missed Same-Day Opportunities

**DOI:** 10.7759/cureus.95263

**Published:** 2025-10-23

**Authors:** George Bechir, Mariam Ameeruddin

**Affiliations:** 1 Hospital Medicine, Franciscan Health Munster, Munster, USA; 2 Biology, Indiana University Northwest, Gary, USA

**Keywords:** afternoon discharge, care transitions, discharge barriers, discharge coordinator, discharge planning, discharge rounds, hospital efficiency, hospital throughput, length of stay, patient flow

## Abstract

Hospital discharge processes often fail to capture patients who become medically ready for discharge in the afternoon hours. While morning discharge rounds have become standard practice, they represent only a single assessment point, missing clinical improvements and resolved barriers that occur throughout the day. This gap results in unnecessary overnight stays, increased costs, and reduced bed availability. This narrative review examines literature from 2010 to 2024 on afternoon discharge interventions, discharge timing patterns, and barriers to same-day discharge. Clinical milestones such as laboratory results, consultant clearance, and symptom improvement commonly occur in afternoon hours, yet current workflows fail to capture these opportunities. Structured afternoon reassessment programs can potentially increase same-day discharges through focused protocols. Successful programs share common features: focused reassessment between 2 PM and 3 PM, clear communication protocols, and appropriate patient selection. The discharge coordinator model shows particular promise, with dedicated staff conducting a systematic afternoon review of patients identified as possible discharges during morning rounds. Barriers to implementation include staff resistance, workflow fragmentation, and system limitations around pharmacy and transportation services. Patient populations respond differently to afternoon reassessment, with medical patients awaiting single barriers showing better results than those with complex psychosocial needs. The late-round follow-up model provides a practical framework for systematic afternoon reassessment using existing resources. Core components include a designated afternoon champion, secure messaging for physician communication, and focused review of specific discharge barriers. Implementation should begin with pilot units, expand based on demonstrated success, and comprise track metrics including reassessment rates and successful discharge percentages. Afternoon reassessment protocols focus on acting promptly once barriers are resolved rather than rushing patients. As hospitals face capacity constraints and rising costs, late-round follow-up offers a low-resource, high-impact intervention to capture missed discharge opportunities and improve patient flow.

## Introduction and background

Every afternoon, the same inefficiency repeats across hospital wards. Patients whose cardiac workups return negative, whose infections have responded to antibiotics, and whose post-procedure observation periods have passed without incident all remain in their beds. They are medically ready for discharge, yet they stay another night because morning rounds have concluded, and no systematic process exists for afternoon reassessment.

Morning discharge rounds have become standard practice in most hospitals, effectively coordinating early discharges and reducing length of stay. These structured huddles, where interdisciplinary teams identify and address discharge barriers, have demonstrated measurable benefits. Morning bedside rounds have been shown to improve patient engagement and satisfaction with care [1], while structured discharge initiatives have been associated with shorter length of stay and significantly higher before noon discharge rates [2]. However, this morning-focused approach creates an unintended consequence: discharge decision-making essentially stops after rounds conclude, ignoring clinical developments throughout the remaining day. For clarity, same-day discharge refers to discharge occurring on the calendar day that clinical readiness is achieved, and late-round follow-up refers to a structured afternoon reassessment process to identify and act on that readiness.

The afternoon hours reveal numerous missed opportunities. Laboratory results pending at morning rounds return normal. Consultants complete their evaluations and provide recommendations. Patients demonstrate clinical improvement following morning interventions. Post-procedure observation periods conclude successfully. Yet without systematic reassessment, these developments do not trigger discharge action.

These delays cascade throughout the healthcare system. Emergency departments board patients while potentially available beds remain occupied. Weekend census inflates when Friday afternoon discharge opportunities become Monday discharges. Insurance authorizations obtained in the afternoon may expire unused when discharge is delayed to the next day, sometimes requiring reauthorization. Each unnecessary night exposes patients to risks, including hospital-acquired infections, adverse drug events, and functional decline, particularly among elderly patients [3,4].

The afternoon discharge gap persists due to workflow patterns and resource allocation. After morning rounds, hospitalists disperse to handle admissions and procedures across multiple units. Nursing shifts change during afternoon hours. Case managers focus on preparing for tomorrow's anticipated discharges rather than revisiting today's evolving possibilities. Without a systematic trigger for afternoon reassessment, potential discharge opportunities remain invisible.

Previous attempts to address this gap lack structure and consistency. Some hospitals have implemented "discharge by" targets, setting afternoon goals during morning rounds. However, without systematic follow-up processes, these aspirations frequently go unmet. Individual practitioners may circle back on possible discharges, but this depends on personal initiative rather than reliable systems. Electronic communication tools have created new channels for care coordination, but often lack defined workflows specifically for afternoon discharge reassessment.

Late-round follow-up offers a systematic solution: structured afternoon reassessment specifically identifying patients whose clinical status has evolved since morning rounds. This focused intervention captures discharge opportunities emerging after traditional decision-making, using existing hospital resources like secure messaging systems and electronic health records (EHRs) to coordinate rapid reassessment and discharge execution. Unlike previous afternoon huddles or "discharge by" initiatives, the late-round follow-up model introduces a defined 2 PM to 3 PM reassessment window and a standardized communication process, making it a structured and repeatable approach for capturing same-day discharge opportunities. This review examines the rationale for structured afternoon reassessment, explores implementation strategies used by early adopter institutions, and proposes a practical framework for late-round follow-up as an essential complement to morning discharge rounds.

## Review

Methodology

We conducted a narrative review of the literature examining afternoon discharge reassessment and its impact on hospital length of stay and discharge timing. Searches were performed in PubMed, CINAHL, and Google Scholar for publications between January 2010 and December 2024. Combinations of the following terms were used: “afternoon discharge”, “late rounds”, “PM rounds”, “discharge timing”, “same-day discharge”, “discharge reassessment”, “second look discharge”, and “discharge optimization”. Additional searches combined these with “length of stay”, “hospital throughput”, “discharge barriers”, and “care coordination”.

Studies were included if they evaluated discharge timing patterns in adult acute care hospitals, examined interventions targeting afternoon or late-day discharges, reported outcomes related to discharge timing or length of stay, or described workflows for reassessment after morning rounds. We also included studies analyzing temporal patterns of decision-making, consultant recommendation timing, and barrier resolution throughout the hospital day. Given the limited literature directly addressing afternoon discharge rounds, we expanded our search to include quality improvement reports describing institutional discharge optimization and studies evaluating secure messaging or communication technology for discharge coordination.

We excluded studies limited to pediatric, psychiatric, or rehabilitation facilities. Emergency department boarding studies were excluded unless they specifically addressed inpatient discharge timing. Studies focused solely on weekend discharges without weekday processes were excluded. Editorials and opinion pieces without original data were excluded, though their reference lists were reviewed for potentially relevant sources.

The search identified 287 articles. After removing duplicates and screening titles and abstracts, 76 full-text articles were reviewed. Following the application of inclusion and exclusion criteria, 42 studies were selected: 12 retrospective cohort studies on discharge timing, eight prospective observational studies of interventions, 14 quality improvement reports, five studies on communication technology, and three systematic reviews.

Data extraction focused on discharge timing, barriers to afternoon discharge, components of successful interventions, role definitions for afternoon reassessment, communication methods, and impact on length of stay and throughput. Special attention was given to resource requirements, implementation challenges, and sustainability.

Given the heterogeneity of interventions and the limited number of studies specifically on afternoon discharge reassessment, findings were synthesized narratively around key themes: (i) evidence for afternoon discharge opportunities, (ii) barriers to implementation, (iii) characteristics of successful interventions, and (iv) practical considerations for late-round follow-up design. Evidence was interpreted with greater emphasis on controlled studies compared to single-site quality improvement reports.

Results

Afternoon Discharge Opportunity: Evidence and Scope

The temporal pattern of hospital discharge decisions reveals a striking mismatch between when patients become medically ready and when discharges actually occur. Multiple studies have documented that discharge readiness evolves throughout the hospital day, yet most institutions maintain rigid morning-only decision-making processes.

Discharge timing data from acute care hospitals demonstrates consistent patterns. A multicenter analysis of discharge timing showed that the majority of discharges occurred between noon and 6 PM rather than before noon, suggesting that many patients became ready after morning rounds [2]. Powell et al. found that delays in discharge decision-making contributed significantly to extended length of stay, with administrative and system factors accounting for more delays than clinical issues [5]. These patterns persist across different hospital types and geographic regions.

Clinical milestones that determine discharge readiness frequently occur in the afternoon. Blood culture results, which require 24-48 hours of incubation, often finalize late in the day. Cardiac biomarkers drawn in the emergency department overnight typically result by mid-day. Post-procedure observation periods, whether for cardiac catheterization, endoscopy, or minor surgeries, commonly conclude in the afternoon. Consultant evaluations, often necessary for discharge clearance, tend to cluster later in the day after specialists complete procedure schedules [6].

The phenomenon of clinical improvement throughout the hospital day is well documented. Patients with acute heart failure often show progressive response to diuresis over 24-hour periods. Elderly patients with delirium may show gradual cognitive improvement as underlying medical conditions are treated. Pain control typically stabilizes hours after initiating appropriate analgesia. These improvements, while potentially occurring throughout the day, often go unrecognized without systematic afternoon reassessment [7,8].

Studies examining unnecessary hospital days highlight the scope of missed opportunities. McDonagh et al. found that medical patients experienced unnecessary hospital days, with discharge planning issues being a primary contributor [9]. A cross-sectional study examining appropriate hospital stay found that over 20% of hospital days were inappropriate, with many patients meeting discharge criteria but remaining hospitalized due to process inefficiencies [10].

The financial implications are substantial. Each avoidable hospital day represents not only direct costs but also opportunity costs from occupied beds unavailable for new admissions. With average daily hospital costs exceeding $2,500 for a medical-surgical bed, capturing even a fraction of afternoon discharge opportunities could generate significant savings [11]. Beyond finances, timely discharge when medically appropriate aligns with patient preferences, as studies consistently show that patients prefer recovering at home when safely possible [12].

Emergency department boarding further underscores the importance of afternoon discharges. Research has shown that emergency department boarding times directly correlate with inpatient bed availability and that improving inpatient discharge timing reduces emergency department crowding [13]. Creating afternoon bed availability through late-round follow-up could therefore provide crucial capacity during peak emergency department admission hours.

Collectively, the evidence suggests that substantial opportunity exists for same-day discharge identification in the afternoon hours, but current hospital workflows fail to systematically capture these opportunities.

Barriers to Afternoon Discharge

Despite the clear opportunity for afternoon discharges, multiple barriers prevent hospitals from systematically capturing these patients. Understanding these obstacles is essential for designing effective late-round follow-up interventions.

Workflow fragmentation represents the primary barrier. After morning rounds conclude, the coordinated team disperses. Hospitalists move between admitting new patients, responding to pages, and completing procedures. A time-motion study of resident workflow found that physicians experience frequent interruptions throughout their shifts, underscoring how fragmented afternoon work becomes [14]. This environment makes it difficult to systematically revisit morning discharge decisions. Additionally, the afternoon often brings new admissions from the emergency department, which naturally take precedence over reassessing existing patients [15].

Nursing shift changes create another critical barrier. Most hospitals schedule nursing shift transitions between 3 PM and 7 PM, precisely when afternoon discharge decisions would need to be executed. During handoffs, essential information transfer occurs, but discharge planning often receives less attention than immediate clinical issues [16]. The incoming night shift nurses may be unfamiliar with patients’ discharge readiness, while the outgoing day shift often lacks time to complete discharge teaching before leaving.

Communication gaps between services further complicate afternoon discharges. While EHRs capture data, they rarely trigger real-time alerts when discharge criteria are met. Studies of communication patterns show that critical information, such as consultant recommendations or laboratory results, may go unrecognized for hours [17]. Without active communication loops, afternoon developments are often overlooked until the following morning.

Institutional culture and expectations add invisible but powerful barriers. Many hospitals implicitly assume discharges occur in the morning, with afternoon and evening staff oriented more toward managing acute issues than facilitating discharges [18]. This expectation influences pharmacy schedules, transportation availability, and ancillary services, creating a self-fulfilling cycle in which afternoon discharges are considered abnormal and thus harder to execute.

Resource allocation poses further challenges. Hospital support services often have reduced availability in the afternoon, with staff focused on preparing next-day discharges rather than executing same-day ones. Physical and occupational therapy evaluations may be limited later in the day. Hospital pharmacies face competing priorities in the afternoon from new admissions and urgent requests, which can affect discharge medication preparation [19].

Financial disincentives may also discourage late discharges. Payment models often reimburse by calendar day rather than hour, limiting financial motivation to discharge patients in the afternoon. In addition, concerns about readmission penalties may make providers hesitant to discharge later in the day when follow-up services are less accessible [20].

Patient and family dynamics add another layer of complexity. Families may be unable to arrange transportation until evening, and some patients prefer “one more night” for reassurance even when medically unnecessary [12]. This psychological comfort can delay otherwise appropriate discharges.

Finally, technology limitations persist despite widespread EHR adoption. Most systems lack intelligent discharge alerts, requiring manual chart review to identify readiness. Integration across laboratory, pharmacy, and consultation systems remains fragmented, demanding active navigation rather than automated synthesis [21].

These barriers interact to create a complex web of obstacles that cannot be addressed by simple fixes. Successful late-round follow-up interventions must therefore recognize and systematically address these multifaceted challenges.

These barriers interact to create a complex web of obstacles that cannot be addressed by simple fixes. Successful late-round follow-up interventions must therefore recognize and systematically address these multifaceted challenges. Among these barriers, staff resistance represents one of the most significant yet underrecognized challenges. The following section focuses specifically on this issue and practical strategies for overcoming it within the late-round follow-up framework.

Existing Models and Interventions

While comprehensive late-round follow-up programs remain uncommon, several hospitals have implemented interventions targeting afternoon discharge opportunities with varying degrees of success.

"Discharge by time" initiatives represent the most widespread approach. These programs set target discharge times during morning rounds, typically aiming for discharge by 11 AM or 2 PM. Beck and Gosik implemented a “discharge by noon” initiative that increased before noon discharges from 11% to 24%, though the improvement plateaued after initial gains [22]. However, without a structured afternoon follow-up, many patients with afternoon target times still remained until the next day. Similarly, team-based rounds aimed at early discharge have shown improvements, though only a minority of patients actually left within two hours of their planned target [23].

Some institutions have experimented with afternoon huddles for discharge planning. Reports describe focused 15-minute discussions among charge nurses, case managers, and hospitalists that increased afternoon discharges and modestly reduced average length of stay [24]. The key success factor was keeping discussions narrowly centered on discharge barriers rather than broader clinical updates.

Technology-enabled interventions have also shown promise. Studies of admission/discharge peak times emphasize the importance of real-time information flow, with dashboards and alerts increasingly used to highlight patients meeting discharge criteria. When such systems were implemented, same-day discharge rates improved for patients meeting criteria after noon [25,26].

Pharmacy-driven programs have addressed medication-related afternoon discharge barriers. A systematic review of pharmacist involvement in inpatient care found that proactive medication preparation for “possible today” patients reduced pharmacy delays substantially, from over an hour to just minutes in some settings [27].

In a recent study led by one of the present authors (Bechir and Bechir), hospitals implementing a dedicated discharge coordinator serving as the operational "traffic controller" of patient flow saw measurable improvements in same-day discharge rates [28]. The coordinator focused exclusively on the 2 PM to 3 PM reassessment, systematically checking test results, consultant notes, post-procedure observation periods, and barrier resolution. Once patients met the criteria, the coordinator ensured all operational tasks (pharmacy, transportation, instructions) were executed promptly. This eliminated many preventable delays that occurred when discharge tasks lacked clear ownership.

The hospitalist “swing shift” model represents another approach, with overlapping coverage in the afternoon hours. Reports indicate that this structure can support discharge reassessment without competing morning duties, improving throughput, though sustainability remains a concern [29].

Nurse-led discharge coordination programs have also shown benefit. Designated discharge nurses conducting afternoon reassessment were most successful when empowered to initiate logistical tasks such as scheduling follow-up appointments and arranging transportation [30].

"Pull" systems provide another example, where post-acute facilities actively engage with hospitals regarding patient transfers. Research on transitions to skilled nursing facilities highlights the complex coordination required between hospitals and post-acute settings, particularly for vulnerable populations such as patients with dementia, where both patient-level and system-level factors must align for successful transitions [31].

Finally, multidisciplinary initiatives have shown promise in improving discharge timing. Kane et al. implemented a structured approach involving multiple disciplines working together to increase inpatient discharges before noon, demonstrating that coordinated team efforts can improve discharge efficiency [32].

Despite these innovations, most interventions remained pilot projects or unit-specific initiatives rather than hospital-wide systems. Programs that succeeded long-term shared key characteristics: brief focused discussions, clear role definition, technology support, and accountability. However, no single model has emerged as standard, underscoring the need for a more comprehensive framework like late-round follow-up.

Addressing Staff Resistance

Healthcare staff's resistance to implementing afternoon reassessment rounds represents a significant barrier. Studies examining new rounding initiatives reveal that staff across disciplines often resist additional rounds due to workflow concerns and competing priorities [33].

Physician resistance typically centers on the perception that afternoon reassessment duplicates morning efforts and interferes with other responsibilities. Greysen et al. found that hospitalists viewed additional discharge-related tasks as burdensome when they were already managing new admissions and acute care needs [33]. However, late-round follow-up differs from traditional rounds by using brief secure messaging rather than physical meetings and by focusing only on patients already identified as potential discharges rather than reviewing all patients.

Nursing resistance often arises from concerns about being pulled away from bedside care for another meeting. Research on shift-change processes shows that nurses are already balancing high-stakes communication demands in the late afternoon [16]. Late-round follow-up addresses this by not requiring bedside nurses to leave patients; instead, charge nurses or discharge coordinators can provide updates while bedside nurses continue direct care.

Case managers and social workers may resist afternoon reassessment, believing that discharge barriers identified in the morning cannot be resolved by afternoon. They often feel overwhelmed with the intensity of morning discharge planning and may view additional afternoon follow-up as unrealistic. Research on transitions of care also highlights the importance of caregiver and family readiness in the discharge process, showing that activation and engagement are key to successful transitions [34]. Evidence demonstrating that specific barriers (lab results, consultant clearance, symptom improvement) frequently resolve by the afternoon helps address these concerns.

Successful implementation requires demonstrating that late-round follow-up actually saves time by preventing next-day rework. Programs that tracked time spent on repeated morning reviews of the same patients who could have been discharged the previous afternoon showed net time savings [6]. Transparent sharing of these efficiency gains helps convert skeptics into supporters.

Special Populations Considerations for Late-Round Follow-Up

The late-round follow-up model requires modification for specific patient populations based on their unique clinical trajectories and discharge complexities.

Elderly patients present distinct considerations for afternoon reassessment. Research on geriatric hospital care demonstrates that older adults often experience slower clinical improvement and require more complex discharge coordination than younger patients [4]. However, studies of geriatric care models show that elderly patients with single acute issues (such as urinary tract infections, mild dehydration, or medication adjustments) superimposed on stable chronic conditions can benefit from afternoon reassessment when their acute issue resolves. The key is distinguishing between complexity that requires time versus issues that simply need confirmation of improvement.

Surgical patients follow protocol-driven recovery pathways that align well with afternoon reassessment. Enhanced recovery after surgery (ERAS) protocols establish objective discharge criteria including pain scores, ambulation distance, and oral intake tolerance [35]. Patients meeting these criteria after morning procedures can be identified through late-round follow-up for same-day discharge. Studies show that standardized postoperative protocols enable safe afternoon discharge for procedures like laparoscopic cholecystectomy and hernia repair when specific milestones are met [35].

Patients with behavioral health comorbidities require careful stratification. Research on psychiatric readmissions emphasizes that rushed discharge planning increases return visits [36]. However, the late-round follow-up model can effectively serve medically admitted patients with stable psychiatric conditions. For example, a patient with controlled bipolar disorder admitted for pneumonia can undergo afternoon reassessment for their medical issues while their psychiatric condition remains stable. The distinction lies in whether the psychiatric condition is the primary barrier to discharge or an incidental comorbidity.

Social complexity significantly impacts late-round follow-up effectiveness. Studies of discharge disparities show that patients with limited English proficiency, homelessness, or lack of social support require extensive coordination that cannot be expedited through afternoon reassessment [12]. These patients benefit more from comprehensive morning discharge planning with next-day execution rather than attempts at same-day resolution. Programs should screen for social complexity during morning rounds to avoid inappropriate afternoon reassessment attempts.

Medical patients generally show the best response to late-round follow-up. Analysis of discharge patterns demonstrates that medical conditions such as heart failure exacerbations, chronic obstructive pulmonary disease (COPD) flares, and cellulitis often follow predictable improvement trajectories amenable to afternoon reassessment [2]. These patients frequently await single rate-limiting steps (final laboratory results, completion of observation periods) that resolve predictably by afternoon, making them ideal candidates for the model.

Discussion

The evidence reviewed highlights a persistent inefficiency: hospitals miss substantial discharge opportunities in the afternoon. While morning rounds have become structured and effective, systematic reassessment later in the day remains largely absent. As a result, patients who are medically ready often remain hospitalized unnecessarily, while emergency departments board new admissions awaiting those same beds.

Proposed Framework: Late-Round Follow-Up Model

The late-round follow-up model provides a practical solution to this gap. Rather than creating a resource-intensive intervention, it leverages existing staff and communication tools in a focused manner. The 2 PM to 3 PM reassessment window aligns with the natural timing of clinical improvement, test result availability, and consultant input.

What makes this model particularly compelling is its simplicity. A discharge coordinator or charge nurse can review the morning “possible discharge” list, verify updated results, send a brief secure message to the attending physician, and coordinate execution of discharge tasks once clearance is granted. Programs that maintained this streamlined approach demonstrated greater sustainability than those requiring new meetings or elaborate processes [28].

Staff resistance to afternoon reassessment is understandable. Afternoons are already filled with admissions, procedures, and competing clinical demands, and additional tasks may feel burdensome. However, evidence shows that structured reassessment ultimately saves time by preventing repeated morning reviews of patients who could have been discharged the previous afternoon [6]. Demonstrating these efficiency gains, rather than mandating participation, appears critical for securing staff engagement.

Appropriate patient selection is equally important. Medical patients awaiting discrete milestones such as negative cardiac markers, completion of observation periods, or consultant clearance are ideal candidates for afternoon reassessment. In contrast, patients requiring complex, multidisciplinary discharge planning with equipment, training, or family coordination benefit more from comprehensive morning planning. Attempting to rush these discharges in the afternoon risks frustration and poor outcomes.

This review also acknowledges the limitations of current evidence. Most studies are single-site quality improvement projects with heterogeneous designs, limiting generalizability. Large multicenter trials comparing standardized afternoon reassessment protocols to usual care are lacking. Publication bias likely favors successful interventions, while failed initiatives may remain unreported. Furthermore, implementation feasibility varies by institutional culture, resources, and patient populations.

Despite these limitations, findings across diverse settings consistently demonstrate that systematic afternoon reassessment identifies discharge opportunities that would otherwise be missed. The specifics of implementation may differ, but the principle remains: hospitals that deliberately reassess patients in the afternoon reduce unnecessary hospital days, improve throughput, and better align discharge timing with patient readiness.

## Conclusions

Hospitals face a fundamental choice: accept that patients identified as "possible discharge" in the morning will remain overnight, or implement systematic afternoon reassessment to capture those who become ready later in the day. Every afternoon, patients meet discharge criteria but remain hospitalized simply because no structured process exists to reassess them. Laboratory results finalize, consultants provide clearance, and observation periods conclude, yet these opportunities are missed while beds remain occupied. This represents not a clinical shortcoming but an operational gap that late-round follow-up directly addresses through a simple model: between 2 PM and 3 PM, a designated individual reviews the morning's possible discharges, checks for updates, communicates with physicians through secure messaging, and executes discharge tasks once approval is obtained.

Implementation challenges, including staff skepticism, workflow integration, and system barriers such as pharmacy and transportation, must be managed, yet hospitals that sustained the process consistently demonstrated durable improvements in length of stay without compromising quality or safety. Afternoon reassessment is not about rushing patients but about acting promptly once barriers have been resolved, meeting the needs of patients who prefer to return home when ready, families who want timely reunification, and hospitals that require the capacity. As health systems confront mounting capacity constraints and rising costs, afternoon discharge opportunities cannot be ignored, as each unnecessary night represents wasted resources and unavailable beds. The path forward is clear: implement structured afternoon reassessment, evaluate outcomes, and expand based on success. The question is not whether to adopt late-round follow-up, but how quickly we can spread this practice to capture the thousands of missed discharge opportunities that occur every afternoon across our healthcare system.
